# Optogenetic inhibition of medial entorhinal cortex inputs to the hippocampus during a short period of time right after learning disrupts contextual fear memory formation

**DOI:** 10.1186/s13041-020-00719-w

**Published:** 2021-01-06

**Authors:** Min Soo Kang, Jin-Hee Han

**Affiliations:** Department of Biological Sciences, KAIST Institute for the BioCentury, Korea Advanced Institute of Science and Technology, Daejeon, 34141 Korea

**Keywords:** Medial entorhinal cortex, Hippocampus, Contextual fear conditioning

## Abstract

Formation of temporal association memory and context-specific fear memory is thought to require medial entorhinal cortex (MEC) inputs to the hippocampus during learning events. However, whether the MEC inputs are also involved in memory formation during a post-learning period has not been directly tested yet. To examine this possibility, we optogenetically inhibited axons and terminals originating from bilateral dorsal MEC excitatory neurons in the dorsal hippocampus for 5 min right after contextual fear conditioning (CFC). Mice expressing eNpHR3.0 exhibited significantly less freezing compared to control mice expressing EGFP alone during retrieval test in the conditioned context 1 day after learning. In contrast, the same optogenetic inhibition of MEC inputs performed 30 min before retrieval test did not affect freezing during retrieval test, excluding the possibility of non-specific deleterious effect of optical inhibition on retrieval process. These results support that contextual fear memory formation requires MEC inputs to the hippocampus during a post-learning period.

The hippocampus-entorhinal cortex system is crucial for episodic memory formation [1]. Evidence suggests that input from medial entorhinal cortex layer III (MECIII) to the hippocampus, mainly to CA1 subfield, is crucial for temporal associative learning such as trace fear conditioning but not for contextual and delayed fear conditioning [2, 3]. In contrast, optogenetic inhibition of medial entorhinal cortex layer II (MECII) cells projecting to dentate gyrus (DG) and CA3, but not cells projecting to CA1, during CFC impairs memory formation [4]. Thus, the input from MECII to DG or CA3 is thought to be required for contextual fear learning. Not only in learning, MEC input to the hippocampus is also involved in unique physiological activity patterns observed in the hippocampus during rest and sleep such as ripples and dentate spikes that are associated with memory consolidation process [5–10]. Therefore, MEC input to hippocampus may have a role for memory formation during a post-learning period. However, this possibility has not been tested yet.

To test whether MEC input to hippocampus is necessary during post-learning period, we adopted an optogenetic approach to silence MEC input activity in 129/C57Bl/6 hybrid mice [11, 12]. To manipulate MEC input, adeno-associated virus (AAV) containing genes encoding eNpHR3.0 fused with fluorescent protein EYFP under the CaMKIIα promoter (AAV-CaMKIIα-eNpHR3.0-EYFP) was injected in the bilateral MEC targeting to layer II and III (0.5 µL per side; AP: – 4.65 mm; ML: ± 3.4 mm; DV: – 3.3 mm) (Fig. 1a). As a control, we injected AAV lacking eNpHR3.0 (AAV-CaMKIIα-EGFP), which only expresses the fluorescent protein. AAV expression was restricted to MEC layers II and III, which sends projection to the hippocampus (Fig. 1b). In the hippocampus, MEC axonal projections expressing EYFP were detected in DG and CA3, originating from MECII, and also in CA1, originating from MECIII (Fig. 1a). Three weeks after the AAV injection surgery, mice were again anesthetized for optic fiber implant surgery. Optic fibers were implanted in the dorsal hippocampus (AP: − 2.0 mm; ML: ± 1.3 mm; DV: − 1.5 mm) for optical suppression of dorsal MEC inputs with a 561-nm laser. Optic fiber tips were located right above CA1 stratum lacunosum-moleculare layer, and therefore, 561-nm laser could cover both MECII afferents in DG molecular layer/CA3 stratum radiatum, and MECIII afferents in CA1 stratum lacunosum-moleculare layer (Additional file 1: Fig. S1). One-week recovery period after the implant surgery, mice went through fiber habituation for 3 days. They were connected to light-delivering optic patch cord and placed in the habituation cage for 5 min without light delivery. After 3 days of habituation, EGFP and eNpHR3.0 mice were trained for CFC. Mice entered the conditioning chamber and received a footshock (0.5 mA, 2 s) 3 min later. One min after the footshock, mice were immediately transferred to the habituated cage and received continuous 561-nm light (5 mW at the fiber tip) for 5 min (Fig. 1b). After post-learning inhibition, mice returned to their home cages. To test whether contextual memory was properly formed, long-term memory (LTM) was tested. Mice re-entered the conditioning chamber 24 h after CFC. Freezing levels were measured as an index of LTM, monitored for 3 min. Mice in eNpHR3.0 group displayed significantly less freezing compared to mice in EGFP control group (Fig. 1c), indicating 24-h LTM deficit by post-learning inhibition of MEC inputs to the hippocampus. This reduction of freezing was not due to a non-specific deleterious effect of optical inhibition process. When the same optical inhibition was delivered 30 min before LTM test, there was no significant difference in freezing between groups (Fig. 1d, e).

**Fig. 1:**
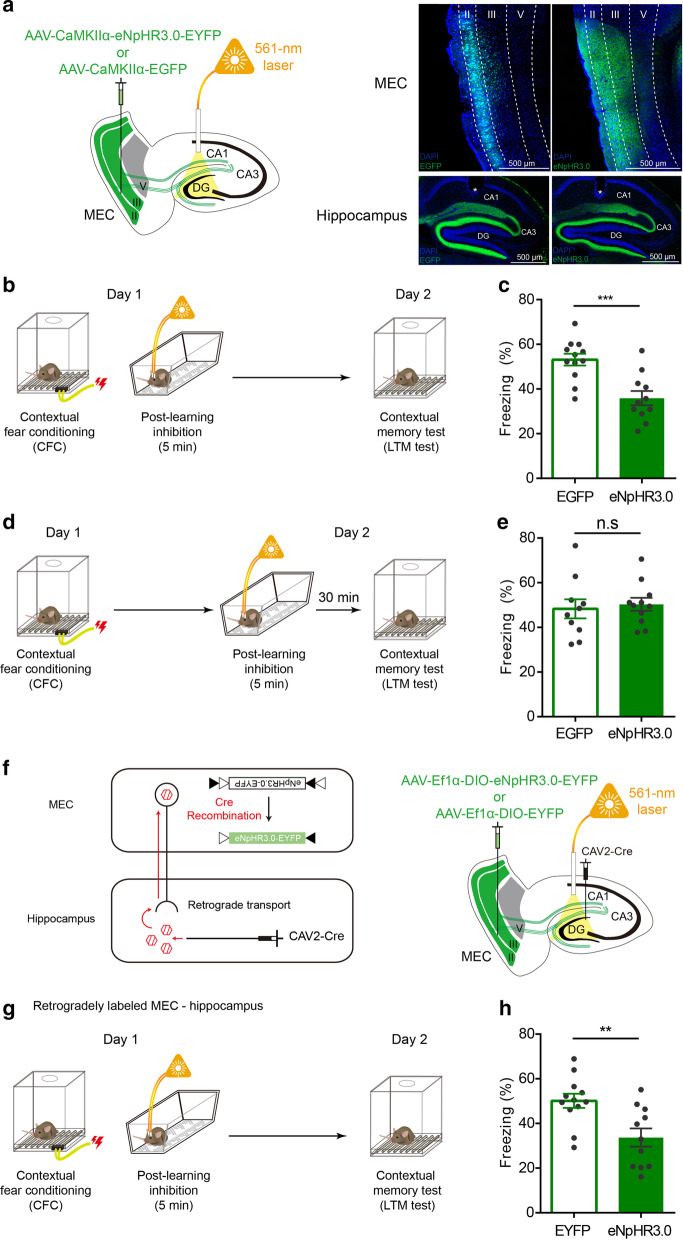
Inhibition of MEC input to hippocampus immediately after learning impairs long-term contextual fear memory formation.** a** Schematic depiction of bilateral AAV injection and optic ferrule implant site (left). Representative confocal microscopic pictures showing EGFP and eNpHR3.0-EYFP expression in MEC (top) and in the hippocampus (bottom). Asterisk indicates approximate position of the optic fiber tip. **b** Behavior scheme. **c** Histogram showing freezing level during test. eNpHR3.0 group (35.89 ± 3.21%, n = 11) showed significantly less freezing level compared to EGFP control group (53.13 ± 2.65%, n = 12) in LTM test. ***p < 0.0005; Student’s *t*-test. **d** Behavior scheme. **e** Histogram showing freezing level during test. eNpHR3.0 (50.31 ± 2.90%, n = 11) and EGFP (48.31 ± 4.27%, n = 10) group showed similar freezing level in LTM test. *p* > 0.05; Student’s *t*-test. n.s, not significant. **f** CAV and AAV injection strategy for retrograde labeling of MEC neurons projecting to hippocampus (left). Schematic depiction of bilateral CAV injection, AAV injection, and optic ferrule implant site (right). **g** Behavior scheme. **h** Histogram showing freezing level during test. eNpHR3.0 group (33.71 ± 4.06%, n = 11) showed significantly less freezing level compared to EYFP control group (50.13 ± 3.18%, n = 12) in LTM test. **p < 0.005; Student’s *t*-test. Data are presented as mean ± SEM

Though majority of MEC afferent targets hippocampus, MEC is known to send projections to medial prefrontal cortical areas such as prelimbic and infralimbic cortex [13]. Since AAV expression was random in MEC, it is possible that our optical inhibition affected the MEC axons that pass through hippocampus, if any, to produce its effect on contextual fear memory formation. To exclude this possibility, we retrogradely labeled MEC neurons that project to hippocampus. We injected CAV2-Cre virus in the hippocampus, which is retrogradely uptaken by neurons that project to hippocampus through axon terminals (Fig. 1f). AAV-Ef1α-DIO-eNpHR3.0-EYFP or AAV-Ef1α-DIO-EYFP was injected into the MEC, so MEC neurons that has uptaken CAV2-Cre can express eNpHR3.0-EYFP or EYFP (Additional file 1: Fig. S2). Then, we managed to inhibit MEC inputs in hippocampus right after CFC. Similar to the prior results, optical inhibition disrupted context fear memory formation (Fig. 1g, h) in this condition. Thus, these data further strengthen our conclusion that MEC inputs to the hippocampus is crucial for contextual memory formation during a post-learning period.

One limitation in this study is the lack of spatial specificity in used optical inhibition. Thus, it is unclear to what extent and exactly where within the hippocampal circuit the widespread MEC inputs were suppressed with the optical inhibition in our condition. Precise inhibition of MEC inputs targeting a specific subfield such as CA1 or DG of hippocampus might be necessary to elucidate the mechanism by which MEC inputs contribute to formation of contextual fear memory in the future study.

Although we did not test the effect of optical inhibition of MEC inputs to the hippocampus at different time points after contextual fear conditioning in this study, previous studies report a time-dependent role of post-learning brain activities for consolidation of hippocampus-dependent memories like contextual fear conditioning. For instance, using an optogenetic strategy, a prior study artificially manipulated the induction of slow-wave sleep (SWS) at different time points following different learning tasks and it appeared that there is a critical time-window (within 30 min) during which SWS induced by light can improve hippocampal-dependent memories [14]. Interestingly, another study in rats showed that selective electrolytic lesions of the direct entorhinal projection (temporoammonic, TA) to the hippocampal area CA1 24 h but not 3 weeks after Morris water maze training disrupted formation of spatial memory at remote time, suggesting that MEC-hippocampus interaction through TA input pathway during this period of time is required for systems consolidation [15]. Given these findings, it is highly likely that MEC inputs to the hippocampus have a time-dependent role for formation of recent and remote contextual fear memory.

It is interesting in our study that such a short period (5 min) of post-training optogenetic inhibition was enough to impair context memory formation. One interpretation is that our results may reflect a partial block of consolidation events that are required for normal memory formation. Consistent with this account, memory was partially impaired in our condition. Another possibility is that there might be a critical event occurring during this brief time point required for formation of contextual fear memory. One possibility is that MEC input may be crucial for triggering ripples or dentate spikes in the hippocampus that are correlated with replay of previously experienced events [7, 9, 10, 16] and optogenetic inhibition of MEC input might disrupt such activity patterns. Alternatively, brief inhibition of MEC input may have affected subsequent slow-wave sleep, which also has a crucial time-window in terms of hippocampus-dependent memory consolidation [14].

## Supplementary Information

**Additional file 1:** **Fig. S1. **Histological verification of optic fiber position in hippocampus.** Fig. S2. **Microscopic images of retrogradely-labeled MEC input to hippocampus.

## Data Availability

The datasets generated and analyzed during the current study are available from the corresponding author upon reasonable request.

## Abbreviations

MEC: Medial entorhinal cortex
DG: Dentate gyrus
AAV: Adeno-associated virus
CFC: Contextual fear conditioning
LTM: Long-term memory
