# Supplementary material for: Optogenetic inhibition of medial entorhinal cortex inputs to the hippocampus during a short period of time right after learning disrupts contextual fear memory formation
Source: Mol Brain. 2021 Jan 6;14:2. doi: 10.1186/s13041-020-00719-w (PMC7789538; doi:10.1186/s13041-020-00719-w)
Supplement: Supplementary file 1 — Additional file 1: Fig. S1. Histological verification of optic fiber position in hippocampus. Fig. S2. Microscopic images of retrogradely-labeled MEC input to hippocampus. [file 13041_2020_719_MOESM1_ESM.docx]

**Additional Files**


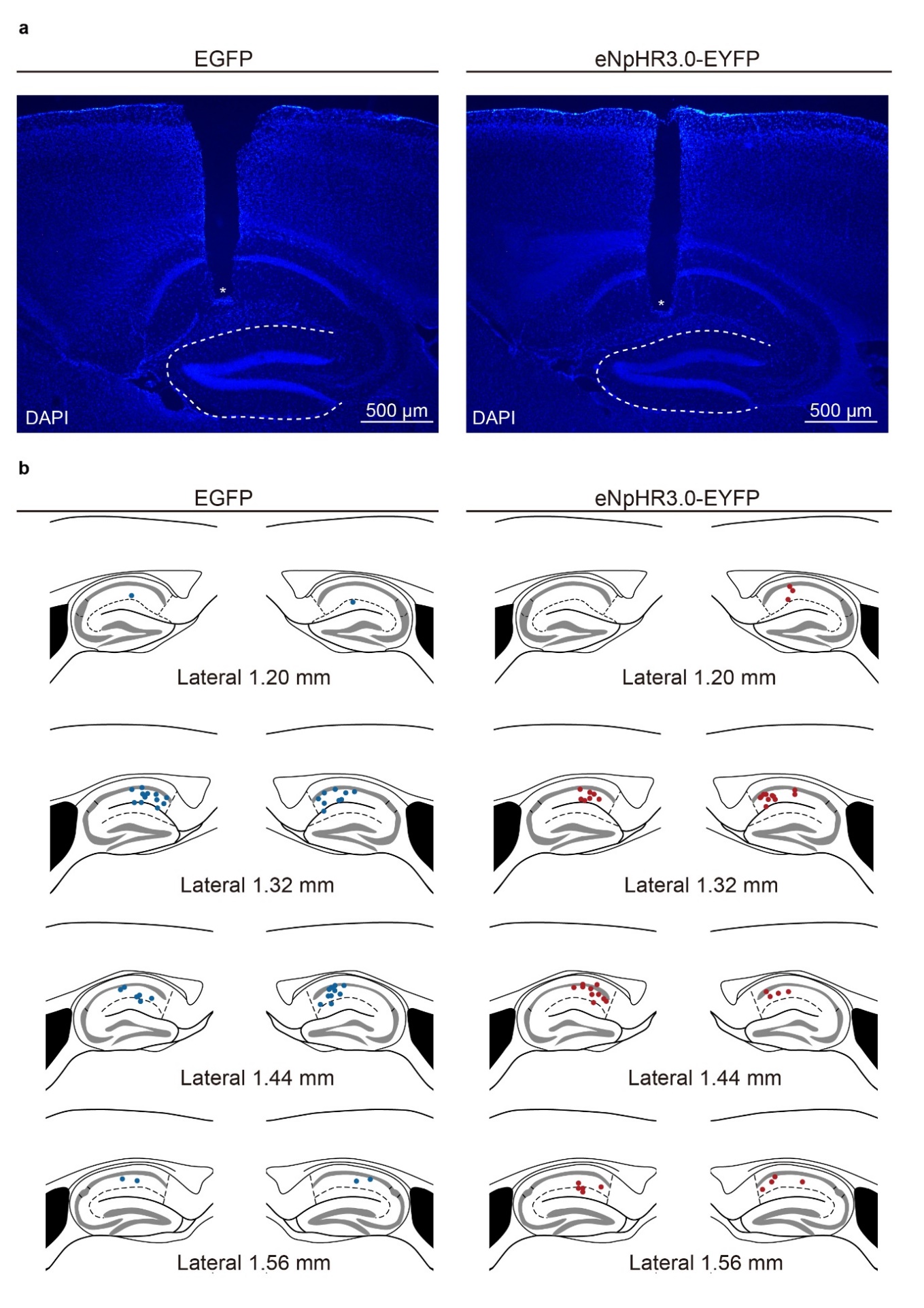


**Fig. S1. Histological verification of optic fiber position in hippocampus.**

**a**) Representative sagittal brain section of EGFP (left) or eNpHR3.0-EYFP (right) expressing section stained with DAPI. Asterisk indicates approximate position of the optic fiber tip. Dotted line indicates the hippocampal fissure. **b**) Schematic illustration of optic fiber position of all mice used in the experiments (left: EGFP, n=22; right: eNpHR3.0, n=22).


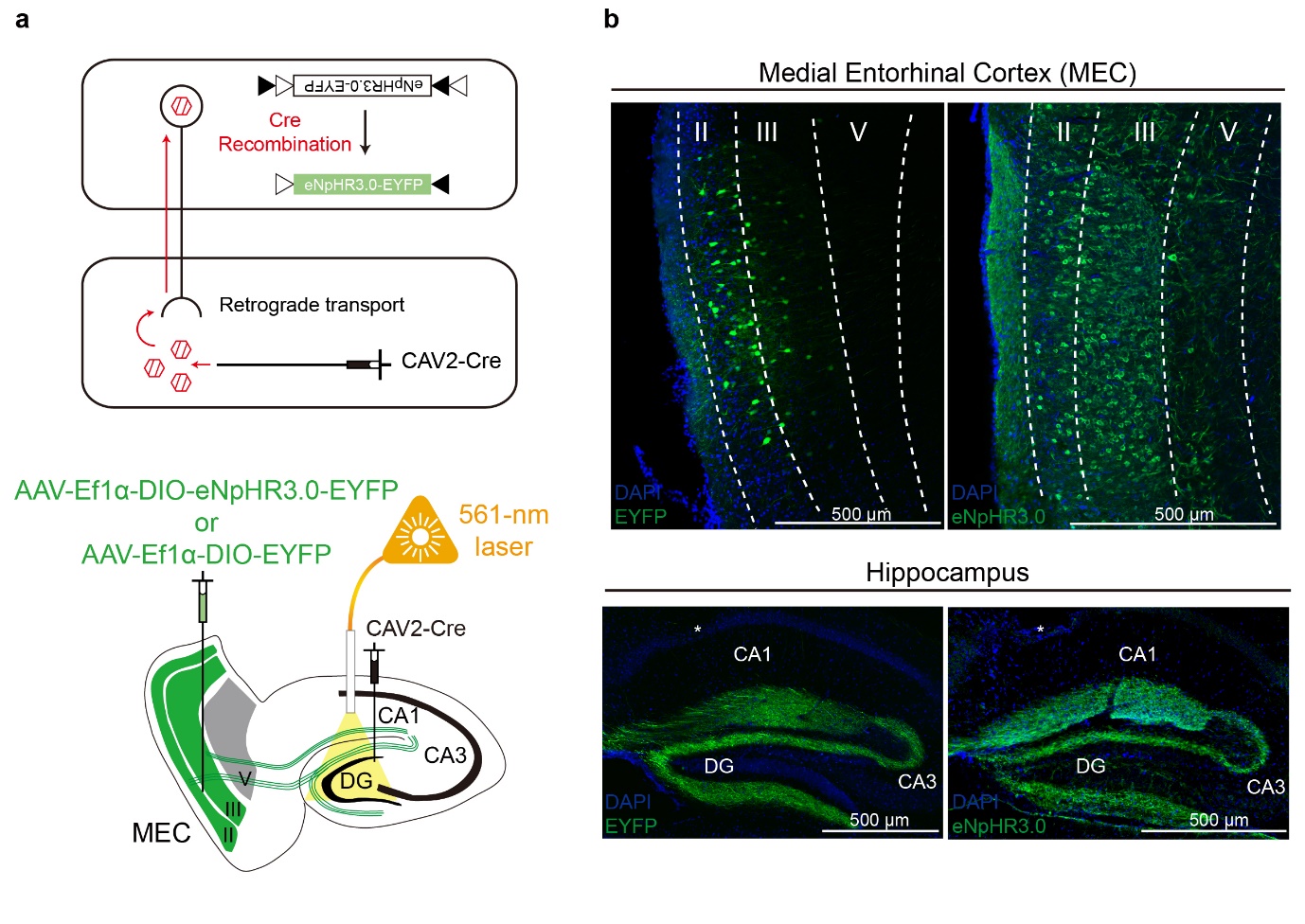


**Fig. S2. Microscopic images of retrogradely-labeled MEC input to hippocampus.**

**a)** CAV and AAV injection strategy for retrograde labeling of MEC input to hippocampus (top). Schematic depiction of bilateral CAV injection, AAV injection, and optic ferrule implant site (bottom). **b)** Representative confocal microscopic pictures showing EYFP and eNpHR3.0-EYFP expression in MEC (top) and in the hippocampus (bottom). Asterisk indicates approximate position of the optic fiber tip.
